# *Sauropus androgynus* (L.) Merr. Induced Bronchiolitis Obliterans: From Botanical Studies to Toxicology

**DOI:** 10.1155/2015/714158

**Published:** 2015-08-27

**Authors:** Hamidun Bunawan, Siti Noraini Bunawan, Syarul Nataqain Baharum, Normah Mohd. Noor

**Affiliations:** ^1^Institute of Systems Biology, Universiti Kebangsaan Malaysia, 43600 Bangi, Selangor Darul Ehsan, Malaysia; ^2^Biotechnology Research Centre, Malaysian Agricultural Research and Development Institute, P.O. Box 12301, 50774 Kuala Lumpur, Malaysia

## Abstract

*Sauropus androgynus* L. Merr. is one of the most popular herbs in South Asia, Southeast Asia, and China where it was known as a slimming agent until two outbreaks of pulmonary dysfunction were reported in Taiwan and Japan in 1995 and 2005, respectively. Several studies described that the excessive consumption of *Sauropus androgynus* could cause drowsiness, constipation, and bronchiolitis obliterans and may lead to respiratory failure. Interestingly, this herb has been used in Malaysia and Indonesia in cooking and is commonly called the “multigreen” or “multivitamin” plant due to its high nutritive value and inexpensive source of dietary protein. The plant is widely used in traditional medicine for wound healing, inducing lactation, relief of urinary disorders, as an antidiabetic cure and also fever reduction. Besides these medicinal uses, the plant can also be used as colouring agent in food. This review will explore and compile the fragmented knowledge available on the botany, ethnobotany, chemical constitutes, pharmacological properties, and toxicological aspects of this plant. This comprehensive review will give readers the fundamental, comprehensive, and current knowledge regarding *Sauropus androgynus* L. Merr.

## 1. Introduction

*Sauropus androgynus* L. Merr. is a shrubby plant belonging to the Euphorbiaceae family. It grows in humid, high temperature conditions and is a Southeast Asian indigenous vegetable, widely cultivated for traditional medicinal purposes.* S. androgynus* is known as Star gooseberry, Sweet leaf bush, Phak waan baan in Thailand, Cekur manis in Malaysia, Katuk in Indonesia, Binahian in Philippines, and Dom nghob in Cambodia.

In Malaysia, this plant is used in traditional medicine to relieve fever, treat urinary problems, and increase breast milk production and consumed as salad, prepared as curry, or stir-fried. It is known as “multigreen” vegetable due to its perceived superior nutrition and vitamin content in comparison to other vegetables [1, 2].* S. androgynus* was reported to have approximately 7.4 g protein per 100 g of fresh leaves whilst, for comparison, spinach has 2.0 g, mint 4.8 g, and cabbage about 1.8 g [1]. The vitamin and mineral composition in* S. androgynus* is summarized in Table 1.

Despite its use as a medicinal and food product, several studies have reported pulmonary dysfunction as a side effect of consuming* S. androgynus*. The first study on the toxic effects of* S. androgynus* was conducted by Bender and Ismail [3]. The study found that unnecessary ingestion of this plant by the elderly in Malaysia caused drowsiness and constipation, demonstrating that the fresh leaves of* S. androgynus* contain 580 mg of alkaloid “papaverine” per 100 gram, and an excessive amount considering only 200 mg of papaverine per day is required as an antispasmodic drug [1, 3]. The first details of problems caused by excessive consumption of* S. androgynus* occurred in Taiwan in 1995 after* S. androgynus* was introduced as supplement to reduce weight in 1994 [4]. Cases of difficulty of breathing were reported, as well as irreversible respiratory failure and death following ingestion of* S. androgynus*. Histopathological examinations found that those patients were diagnosed with constrictive bronchiolitis obliterans and lung transplantation was the only way to treat the condition [5–9].

Due to the impact of this plant on the human consumption, this review will focus on collating the fragmented information regarding botany, chemical composition, pharmacological effects, and toxicology of* S. androgynus*, highlighting the current state of knowledge and providing an intensive overview to readers and researchers.

## 2. Botany

### 2.1. Botanical Name

It is called* Sauropus androgynus* (L.) Merr.

### 2.2. Synonyms

*Aalius androgyna* (L.) Kuntze;* Aalius lanceolata* (Hook.f.) Kuntze;* Aalius oblongifolia* (Hook.f.) Kuntze;* Aalius retroversa* (Wight) Kuntze;* Aalius sumatrana* (Miq.) Kuntze;* Agyneia ovata* Poir.;* Andrachne ovata* Lam. ex Poir.;* Clutia androgyna* L.;* Phyllanthus strictus* Roxb.;* Sauropus albicans* Blume;* Sauropus albicans* var.* gardnerianus* (Wight) Müll.Arg.;* Sauropus albicans* var.* intermedius* Müll.Arg.;* Sauropus albicans* var.* zeylanicus* (Wight) Müll.Arg.;* Sauropus convexus* J.J.Sm.;* Sauropus gardnerianus* Wight;* Sauropus indicus* Wight;* Sauropus lanceolatus* Hook.f.;* Sauropus oblongifolius* Hook.f.;* Sauropus parviflorus* Pax & K.Hoffm.;* Sauropus retroversus* Wight;* Sauropus scandens* C.B.Rob.;* Sauropus sumatranus* Miq.;* Sauropus zeylanicus* Wight [10].

### 2.3. Botanical Description and Distribution

**Figure 1:**
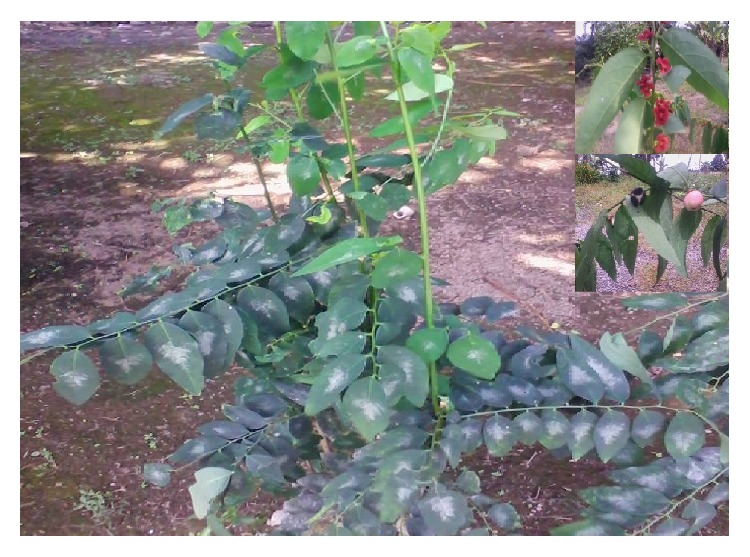
*Sauropus androgynus* L. Merr., also known to Malaysian as Cekur Manis.

*Sauropus androgynus* is an erect shrub that can reach up to 500 cm in height. It is flaccid and has cylindrical or angled branches (Figure 1). The leaves are ovate or lance-shaped, measuring 2.0–7.5 cm and obtuse or acute. The male flowers are disk-shaped, entirely or nearly so. The fruit is nearly globular, up to 1.5 cm in diameter and whitish [10]. This plant is widely cultivated and native to Southeast Asia including Bangladesh, India, Guangdong, Guangxi, Hainan, and Yunnan [10].

## 3. Ethnobotanical Uses

*Sauropus androgynus* has been known as “multigreen” due to its high vitamin and nutrient content and this vegetable is usually consumed raw in salad, stir-fried, used in curry, or cooked in soups in most countries in Southeast Asia. Also,* S. androgynus* is believed to increase lactation in women in Indonesia and Malaysia [11, 12]. Furthermore, Thai people traditionally use the roots of this plant to reduce fever and treat food poisoning and as antiseptic agent [1, 13]. People in Taiwan believe that* S. androgynus* could have significant potential as a slimming agent to combat obesity. In India, the leaves of this plant are used as antidiabetic and to improve vision. Traditional uses of* S. androgynus* are summarized in Table 2.

## 4. Chemical Compositions

Phytochemical investigation of the leaves of* S. androgynus* reveals they contained sterols, resins, tannins, saponins, alkaloids, flavonoids, terpenoids, glycosides, phenols, catechol, cardiac glycosides, and acidic compounds [14]. Previously, preliminary phytochemical screening on the leaves of* S. androgynus* showed polyphenols, anthocyanins, carotenoids, ascorbic acids, and tannins [2]. Further phytochemical studies by Gireesh et al. [15] suggested that the leaf ethanol and aqueous extracts of* S. androgynus* contain tannins, saponins, flavonoids, terpenoids, phenolics, steroids, and alkaloids. These preliminary phytochemical studies suggested that* S. androgynus* contains a wide range of biomolecules constituents that might contribute to its medicinal, toxic, and antioxidant properties.

Wang and Lee [16] isolated six compounds (three nucleosides, two flavonol diosides, and one compound flavonol trioside) based on spectral analysis from butanol extract of* S. androgynus* leaves. One of the novel isolated compounds, known as 3-O-*β*-D-glucosyl-(1-6)-*β*-D-glucosyl-kaempferol (GGK), was reported to have a high potential as an antiobesity agent [17]. A study by Kanchanapoom et al. [18] elucidated and identified seven bioactive compounds from the methanolic extract of the aerial part of* S. androgynus*, including three lignan glycosides, a lignan diglycoside, a megastigmane glucoside, and a megastigmane glucoside compound.* S. androgynus* leaves were found to have highest content of flavonoids and bioactive compounds among 11 vegetables from Indonesia with 142.64 mg per 100 gram of fresh weight with quercetin, myricetin, luteolin, apigenin, and kaempferol which were detected by HPLC analysis [12, 19]. Further, phenolic acids such as chlorogenic acid, caffeic acid, and ferulic acid were also identified in their study.

Selvi and Basker [20] found occurrences of phytol and squalene in ethanolic extract of* S. androgynus *using gas chromatography-mass spectrometry (GC-MS) analysis. Further analysis on ethanolic* S. androgynus* leaves extract by GC-MS discovered nine compounds with medicinal functionality such as antimicrobial, anti-inflammatory, antioxidant, and anticancer properties [14].

## 5. Pharmacological Properties

### 5.1. Antidiabetes Activity

Sai and Srividya [21] presented an experiment which focused on the evaluation of the effects of aqueous leaf digest of* S. androgynus* on the postprandial glucose levels in human blood. The results show that glycemic index (GI) scores for patients that were administered the* S. androgynus* leaf digest were significantly lower compared to the control group. This result suggests that this plant possesses a high potential for lowering glucose levels in human blood, which would likely assist in the global battle to reduce diabetes.

### 5.2. Antiobesity Effect

High consumption of young leaves of* S. androgynus* over a long period in the belief that the plant helps to maintain weight resulted in a sudden increase of bronchiolitis obliterans in Taiwan, which is normally rare life-threatening lung disease. The believe that* S. androgynus* had values as an antiobesity agent was confirmed in a study by Yu et al. [17], which focused on effects of* S. androgynus* isolated GGK compound in combination with EtOAc and* n*-BuOH fractions for body weight reduction in Wistar male rats. The results show that 60 mg per kg dose of GGK led to a decrease of food intake of rats by 15% and this led to a reduction in body weight of these rats. This loss of food intake directly corresponded to doses of GGK administered to different groups of rats. Further, no histopathological changes were observed. It was concluded that GGK has a potential to become an antiobesity agent and is unlikely to result in similar side effects as observed when consuming the entire plant.

### 5.3. Wound-Healing Activity

A test demonstrating wound-healing activities of 5% water extract of* S. androgynus* with both male and female rats was reported by Bhaskar et al. [22]. The study found that* S. androgynus* considerably increased contraction of wounds, reepithelization, and wound breaking strength. Histological testing of wound tissue was also reported, and smaller macrophages and fibroblasts, as well as rich collegenation, were observed in comparison to the rat control group. Based on these findings it can be concluded that* S. androgynus* has potential as a wound-healing agent and further research could be conducted to bring this type of produce to market.

### 5.4. Anti-Inflammatory Activity

Anti-inflammatory activity of* S. androgynus* was reported by Senthamarai Selvi and Bhaskar [23]. The study evaluated the effect of ethanolic and aqueous leaf extracts of this herb on Wister rats using carrageenan induced rat paw edema. The ethanolic extract showed a higher anti-inflammatory effect than aqueous extract. This was supported by another study on* in vitro* anti-inflammatory properties of the methanolic extract of this plant, showing significant nitric oxide inhibitory activity using Griess assay [24].

### 5.5. Induction of Lactation Activity

An experiment was performed to verify the effect of* S. androgynus* on production of breast milk as it is commonly consumed for this purpose in Indonesia. House mice (*Mus musculus*) were used for this test and were divided into groups and administered leaf extract of fully grown* S. androgynus* in various dosages [11]. The study found that both expressions of the oxytocin gene and prolactin gene expression levels remarkably increased according to the amount of* S. androgynus* administered. The administration of the extract resulted in smoother circulation of the oxytocin hormone in the mouse bloodstream. Based on the experiment by Soka et al. [11],* S. androgynus* was found to have beneficial effect on breast milk production in mice and could become a breast milk production agent subject to future research.

### 5.6. Antioxidant Activity

Several studies reported positive antioxidant activity of* S. androgynus *[14, 19, 25]. A study conducted by Andarwulan et al. [19] implies potential of this herb to become a strong antioxidant agent. This plant was found to have highest flavonoid content among 11 vegetables of Indonesian origin. Another experiment done by Badami and Channabasavaraj [25] used methanol extracts of this herb using several* in vitro* free radical-scavenging assays. The study found that* S. androgynus* has IC_50_ value of 341 *μ*g/mL, 12.58 *μ*g/mL, and 228.75 *μ*g/mL using DPPH radicals, ABTS cation radicals, and inhibition of lipid peroxidation, respectively. A previous study on antioxidant activities on aqueous extracts of 25 tropical plants showed that* S. androgynus* has a high polyphenol content, cupric ion chelating activities, free radical scavenging, and reducing ferric ion antioxidant properties [26].

### 5.7. Antimicrobial Activity

Methanolic and ethanolic extracts of* S. androgynus* have been reported to have significant antibacterial activity against* Bacillus cereus*,* Proteus vulgaris*, and* Staphylococcus aureus* [27]. On the other hand, aqueous extract demonstrated moderate antibacterial ability. This was later confirmed by Ariharan et al. [28]. The study also found that methanol extract significantly showed increased antibacterial activity compared with aqueous extract against gram positive bacteria. A further study found that ethanol extracts of* S. androgynus* produced higher antibacterial effects against* Klebsiella pneumonia* and* S. aureus* in comparison to aqueous extracts [29]. The extracts of* S. androgynus* were also found to have antifungal inhibitory effects against several fungi such as* Aspergillus flavus* and* Candida albicans* [30].

## 6. Toxicity of* Sauropus androgynus*

*In vitro* cytotoxicity and genotoxicity of this herb were studied by Xin et al. [31] who examined cooked and uncooked leaf juice of* S. androgynus *on CHL cells. Effects of these juices were observed at various concentrations for CHL cells which were exposed to the juice for one to three days. The study found that this herb effects two components inside of the cells (obliteration to lysosomes and golgi apparatus) when administered in certain doses; however, no chromosome changes were perceived as a result of administration of* S. androgynus*.

Further study on the toxicity of this plant has been done by Yu et al. [32], with use of polarity dissection, dividing the extract of* S. androgynus* into three different fractions (*n*-BuOH fraction, CHCI_3_ fraction, and EtOAc). NIH3T3 fibroblasts cells were exposed to these fractions at concentration of 300 *μ*g/mL and tested for toxicity. The results showed that the EtOAc fraction has the leading effect on cell growth inhibition and more importantly that apoptosis and necrosis are present when all the extracts are applied to NIH3T3 fibroblasts. Additional safety assessments on this plant have been conducted using human cell lines MRC-5 and Hep G2 treated with raw* S. androgynus* homogenate at various doses for 24 hours [33]. The homogenate of* S. androgynus* was found to be more toxic on MRC-5 cells (IC_50_: 133.1 mg/mL), the cells derived from human lung compared to Hep G2 cells (IC_50_: 185.1), and cell line derived from liver tissue. In contrast,* S. androgynus* does not contribute to the genetic damage using micronuclei test [33].

Recently, Yunita et al. [34] evaluated methanol leaves extracts of the* S. androgynus* from six different parts of East Java Province, Indonesia, on human mesenchymal stem cells, the cells originating from bone marrow. Surprisingly,* S. androgynus* methanol extract was found to be less cytotoxic to the cells tested with IC_50_ 2450 mg/L. However, the methanol extracts displayed an effect on the viability of the cells that might contribute to cytotoxicity of this plant. To our knowledge, there is no* in vivo* toxicology test that has been conducted to clarify the toxic effects, the period of the effects, and severity and degree of reversibility of this plant. Despite having many positive effects on human health as a multigreen and multivitamin plant, consumption of* S. androgynus* is often connected to bronchiolitis obliterans.

## 7. *Sauropus androgynus* Induced Bronchiolitis Obliterans

The connection between the consumption of the herb and potentially deadly bronchiolitis obliterans (BO) was discovered shortly after* S. androgynus* was introduced in Taiwan as a weight reduction agent in 1994 [4]. An outbreak of* Sauropus androgynus* induced bronchiolitis obliterans was reported in Taiwan in 1995 with approximately 278 patients diagnosed after consumption of uncooked* S. androgynus* [4, 35]. A total of nine patients died and lung transplantation was the only option for treatment of this problem [4, 36, 37]. According to Bender and Ismail [3], leaves of* S. androgynus* contain papaverine as its main toxic component and this compound was found to induce pulmonary disease in animal studies [38]. Wu et al. [5] describe papaverine as a smooth muscle relaxant and vasodilator with recommended intake of 300 mg a day in doses of 150 mg every 12 hours. Those consuming 600 grams of leaves would achieve an intake of 3480 mg weekly. Adverse reactions to the substance are skin rash, abdominal pains, hepatotoxicity, flushing, headache, tachycardia, and dizziness.

Common symptoms of BO disease are firstly palpitation and insomnia, leading to further symptoms which are tightness in chest and dyspnea and many patients were also reported to suffer from respiratory distress. Once these symptoms develop, spirometry and lung biopsy specimens are found to have obstructive ventilatory problems that mean that BO disease was induced. Lai et al. [4] claim that, during a two-year period after having been infected with BO, pulmonary deterioration and even death in 7 of 115 cases (6.1% of patients) occurred. The pathogenesis of* S. androgynus*-associated BO is still unknown. However, it is not an infectious disease. While most patients' status remained stable with cough and dyspnea, about a fifth of them recorded decreasing respiratory function; some even developed a failure and became dependant on ventilators.

Hung et al. [39] demonstrated possible ways of detecting this disease. Methods such as pulmonary function tests and high-resolution computed tomography can help to discover diffuse bronchiectasis as well as mosaic attenuation. With the use of radioaerosol lung scans, signs such as inhomogeneous distribution of aerosol or higher alveolar permeability can be observed. High-resolution computed tomography is a method which has been proven to be very useful with detection of BO and also patchy low attenuation with mosaic perfusion. The main advantage of this method is in fact that it helps to specify this particular disease and prevents it from being mistaken for other respiratory lung diseases. Another useful method is a DTPA lung scan. It was initially used to evaluate lung status of patients after* S. androgynus* intake. A high variety of abnormalities were found which was observed as inhomogeneous aerosol distribution and impaired lung epithelial permeability. This is particularly useful for gathering information about ventilation of lungs; the clearance of DTPA also shows alveolar epithelial permeability.

Many different medications have been administered to patients suffering from* S. androgynus*-induced BO in an attempt to improve their condition. Cytotoxic agents and steroids, as well as other immunosuppressive agents, have been tried in order to reduce the effects of the disease and ease the symptoms. Unfortunately, no patient with the advanced stage of the disease has shown signs of improvement. Patients in advanced stage of the illness usually become dependent on ventilators which are difficult to regulate due to airway obstructions and then patients die due to respiratory failure. Complete lung transplantation seems to be the only solution for patients in advanced stage of the disease [40, 41].

### 7.1. Papaverine: An Alkaloid in* Sauropus androgynus* Responsible for Bronchiolitis Obliterans

Little is known regarding how ingested* S. androgynus* induces bronchiolitis obliterans [42]. In general, the bronchiectasis and mosaic attenuation were present in patients consuming high level of the alkaloid papaverine in* Sauropus androgynus* [5, 7, 40, 43]. Inflammation in small airways and fibrotic lesions of the bronchioles were also observed as well as narrowing of their lumens [44]. Intratracheal papaverine was reported to induce constrictive bronchiolitis obliterans (CBO) in an animal model [42]. On the seventh day after the start of administering papaverine to rats using intratracheal papaverine method, animals started to show common symptoms of CBO. These included extensive denudation, peribronchial inflammation, bronchial mucosa degradation, and an increase in peribronchial collagen [42]. Further, after four weeks of administration of papaverine, the toxicant induced model produced further severe disruption in rats bodies. In papaverine treated animals, two cytokines deregulated in human CBO, TGF-*β* and eNOS, were found at significantly high levels. These cytokines might be responsible for pathogenesis of CBO in humans, which leads to the harmful pulmonary changes connected to CBO.

The study reported by Svetlecic et al. [42] also found that the rats that received papaverine for four weeks had a high level of TGF-*β* in the lung homogenate supernatant. This finding matches with studies that found TGF-*β* immunohistochemically in humans with transplant induced BO [45]. As a marker of airway fibrous obliteration, TGF-*β* induced fibrosis leads to airway constriction and luminal obliteration. Endothelial nitric oxide synthase (eNOS) was also found to be significantly high in rats that received papaverine treatment for 1 to 4 weeks [42]. This enzyme is important for both destruction of the epithelium and stimulation of fibroblast assay in animal model [46]. In humans, it is generally expressed in airway epithelium of human transplant induced CBO. The significant increase of TGF-*β* and eNOS in animal models induced by papaverine suggests that papaverine has the potential to induce bronchiolitis obliterans and this reflects the mechanism observed in humans [42].

## 8. Conclusions

*Sauropus androgynus* is a traditional medicinal plant used in Southeast Asia, especially in Malaysia known as a green multivitamin herb. Previous studies have reported the prospective of this plant as antioxidant, antimicrobial, wound-healing, anti-inflammatory, antidiabetic, and antiobesity agent as well as its potential to increase breast milk production. Despite these pharmacological properties, consumption of its raw leaf juice for weight reduction resulted in outbreak of bronchiolitis obliterans. It seems that papaverine is the chemical compound responsible for occurrence of this pulmonary failure. Further studies should be made in order to fully understand the cytotoxicity of this plant, mechanism of toxicity, dosages and ways of consumption, and effects on human health.

## Figures and Tables

**Table 1:** Nutrition assessment of *Sauropus androgynus* content in 100 g of fresh leaves.

	Value^a^	Value^b^
Protein	7.4 g	5.25 g
Fat	1.1 g	0.58 g
Fibre	1.8 g	1.75 g
Moisture	69.9 g	85.4 g
Carotene	5600 *μ*g	—
Riboflavin	0.21 mg	—
Thiamine	0.50 mg	—
Potassium	—	45.7 mg
Cobalt	—	1.62 mg
Manganese	—	25.6 mg
Copper	—	768.7 mg
Sodium	—	306.3 mg
Zinc	—	15.9 g
Fe	—	212.5 mg
Magnesium	—	664.9 mg
Calcium	711 mg	84.4 mg
Vitamin C	244 mg	314.3 mg
Phosphorus	543 mg	—
Iron	8.8 mg	—

^*∗*^Based on the proximate composition studied by ^a^Padmavathi and Rao [1] and ^b^Singh et al. [2].

**Table 2:** Ethnobotanical uses of *Sauropus androgynus* L. Merr.

Uses	Locality	Reference
A medication for coughs, to soothe lungs, as a tonic to reduce fever, a natural slimming agent, and consumption as a vegetable.	China	[10]

To increase milk production, as an antipyretic, consumed as a vegetable and used as a colorant.	Indonesia	[11, 12, 47]

The roots are used to reduce fever and to reduce food poisoning and as an antiseptic.	Thailand	[1, 13]

The roots are used to treat cardiovascular diseases and hypertension. The leaves are used to increase lactation and for treatment of oral thrush of infants as well as an eye lotion.	Malaysia	[48, 49]

Leaves are used as an antidiabetic, to treat ulcers, eye disease, and tonsillitis.	India	[21, 50]

To improve vision and to treat eye diseases.	Southeast Asia inhabitants	[51]
